# Developmental Regression and Seizures in a 28-Month-Old Child: A Delayed Diagnosis of Anti-N-Methyl-D-Aspartate Receptor Encephalitis

**DOI:** 10.7759/cureus.112687

**Published:** 2026-07-15

**Authors:** Dac Cuong Nguyen, Van Trung Nguyen, Bich Van Nguyen

**Affiliations:** 1 Department of Pediatrics, Rostov State Medical University, Rostov-na-Donu, RUS; 2 Department of Pediatrics, Hai Phong University of Medicine and Pharmacy, Haiphong, VNM; 3 Department of Neurology and Psychiatry, Haiphong Children’s Hospital, Haiphong, VNM

**Keywords:** anti-nmda receptor, encephalitis, normal brain mri, normal cerebrospinal fluid analysis, normal eeg, regression, seizures

## Abstract

Anti-N-methyl-D-aspartate (anti-NMDA) receptor encephalitis is a diagnostic challenge. Delayed treatment may result in irreversible neurological complications and can even be life-threatening if pediatricians do not recognize it early and manage it comprehensively. We report the case of a 28-month-old child whose diagnosis was initially missed, even at a level 1 children’s hospital, because there were no significant changes on brain magnetic resonance imaging (MRI), cerebrospinal fluid analysis (CSF), or electroencephalography (EEG), despite developmental regression and seizures. We conclude that autoimmune encephalitis should be kept in mind even when imaging and other diagnostic tests show no significant abnormalities. Eventually, the patient was successfully treated with immunotherapy. This case highlights the importance of early diagnosis and treatment, as well as the strong correlation between medical history, clinical progression, and diagnostic findings, and the need for suspicion even when initial investigations are inconclusive.

## Introduction

In pediatric neurology practice, diagnosing patients presenting with neuropsychiatric decline can be challenging, as the interval between symptom onset and diagnosis often ranges from several weeks to three months.

Current diagnostic criteria for possible autoimmune encephalitis require subacute onset, usually within a few weeks and less than three months, of altered mental status, working memory deficit, psychiatric manifestations, or other evidence of central nervous system involvement. The criteria also require at least one supportive feature, such as new focal central nervous system findings, seizures not explained by a previous seizure disorder, cerebrospinal fluid (CSF) pleocytosis, or brain magnetic resonance imaging (MRI) findings suggestive of encephalitis. Alternative causes must also be reasonably excluded, and all three criteria must be met [1].

Anti-N-methyl-D-aspartate (anti-NMDA) receptor encephalitis is the most common subtype of autoimmune encephalitis in children and young adults. The second and third most common subtypes are acute disseminated encephalomyelitis, which is associated with myelin oligodendrocyte glycoprotein (MOG) antibodies in approximately 60% of cases, and limbic encephalitis, which comprises several immunological subtypes, with anti-leucine-rich glioma-inactivated 1 (anti-LGI1) encephalitis being the most frequent, usually affecting patients older than 50 years [2].

Risk factors for anti-NMDA receptor encephalitis remain incompletely understood and continue to be controversial.

## Case presentation

Patient information

The child was born full-term by C-section. His birth weight was 3,300 g. The prenatal period was uneventful. The child achieved all developmental milestones appropriately, including walking at around 14 months of age. The only concern was regarding his speech development. His mother noted that he was only cooing at 22 months of age and was able to speak only single-word phrases at around 2 years old. His vocabulary was limited.

Clinical findings

On February 25, one day after Japanese encephalitis (JE) vaccination, he developed stumbling and seizures during sleeping time and during daytime, especially in the right extremities. There were no signs of vasoconstriction or oxygen deficiency. These symptoms lasted until March 10. Fifteen days later, he still had a sign of myoclonus in the right leg.

**Figure 1:**
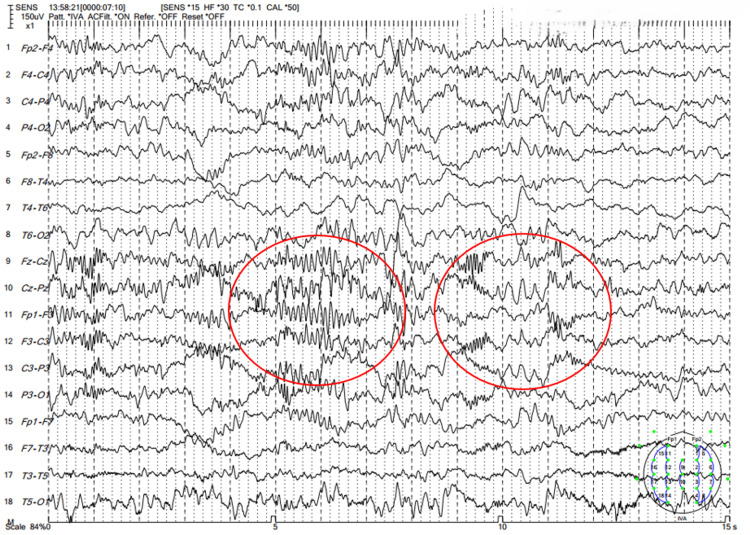
EEG showing spike-and-wave activities (red circles) EEG: electroencephalography

Brain computed tomography revealed no significant changes. When sleeping, electroencephalography (EEG) showed spike-and-wave activities, which were dominant in the left medial frontotemporal area (Figure 1).

This finding was typical since NMDA receptors are distributed dominantly in the frontotemporal area, which undergoes atrophy in this type of encephalitis, leading to changes in behavior [3].

However, at that time, he was diagnosed with focal epilepsy, for which he was prescribed levetiracetam 500 mg/5 mL, 3 mL per day starting March 13.

Twenty-four days later, all symptoms persisted and worsened. The child was unable to speak for three days and unable to walk for four days. There was irritability, mainly at night, and dystonia of the right hand. These symptoms could not be explained by the latest diagnosis of epilepsy.

Medical examination revealed a conscious child with delayed speech, movement disorder of the right hand, negative meningeal signs, and no other remarkable symptoms. The diagnostic assessment is presented in Table 1 and Table 2.

**Table 1:** Diagnostic assessment

Investigation	Result	Interpretation
White blood cell count	6.72 × 10^9^/L	No marked leukocytosis
Neutrophils	26.7%	No neutrophil-predominant inflammatory pattern
Anti-streptolysin O test	Negative	No evidence supporting recent streptococcal immune trigger
Metabolic screening (Table 2)	No abnormalities in amino acid, fatty acid, or organic acid metabolism	Metabolic disease was not supported
Cerebrospinal fluid leukocytes	3 leukocytes/uL	No cerebrospinal fluid pleocytosis
Cerebrospinal fluid protein	0.21 g/L	No marked protein elevation
Cerebrospinal fluid glucose	4.37 mmol/L	No hypoglycorrhachia
Ceruloplasmin	0.23 g/L	No major abnormality reported
Real-time polymerase chain reaction testing for herpesvirus panel	Negative	Herpesvirus infection was not supported
Real-time polymerase chain reaction testing for parvovirus B19, adenovirus, hepatitis E virus, human parechovirus, and mumps virus	Negative	These viral etiologies were not supported
Neuronal antibody panel	Positive only for anti-N-methyl-D-aspartate receptor antibodies	Supported anti-N-methyl-D-aspartate receptor encephalitis
Brain magnetic resonance imaging	No abnormal findings	Normal imaging did not exclude autoimmune encephalitis
Repeat electroencephalography	No spike-and-wave activity	Electroencephalography was nondiagnostic at this stage

**Table 2:** Metabolic disorder screening Interpretation: Based on the provided reference ranges, all listed markers are within the reported normal limits.

Test	Result	Reference range	Unit
Alanine (Ala)	158.27	84.00-649.75	µmol/L
Arginine (Arg)	28.09	1.00-42.42	µmol/L
Aspartic acid (Asp)	19.42	11.37-297.90	µmol/L
Citrulline (Cit)	19.12	4.6-28.0	µmol/L
Glutamic acid (Glu)	199.07	106.00-1313.39	µmol/L
Glycine (Gly)	127.25	55.00-1093.78	µmol/L
Leucine (Leu)	72.61	33.00-283.84	µmol/L
Lysine (Lys)	216.17	79.54-438.00	µmol/L
Methionine (Met)	11.65	4.20-36.00	µmol/L
Ornithine (Orn)	47.22	14.00-325.41	µmol/L
Phenylalanine (Phe)	30.93	17.47-93.33	µmol/L
Proline (Pro)	52.20	36.00-338.66	µmol/L
Serine (Ser)	97.75	47.60-1163.00	µmol/L
Tyrosine (Tyr)	31.47	13.80-260.00	µmol/L
Valine (Val)	91.74	32.2-321.65	µmol/L
Free carnitine (C0)	23.59	8.50-66.12	µmol/L
Acetylcarnitine (C2)	12.35	3.7-52.0	µmol/L
Propionylcarnitine (C3)	1.13	0.19-4.50	µmol/L
Butyrylcarnitine (C4)	0.20	0.00-0.75	µmol/L
C4OH	0.09	0.00-0.33	µmol/L
Isovalerylcarnitine (C5)	0.16	0.00-0.42	µmol/L
Tiglylcarnitine (C5:1)	0.07	0.00-0.09	µmol/L
C5OH	0.34	0.00-0.45	µmol/L
C5DC	0.05	0.00-0.25	µmol/L
Hexanoylcarnitine (C6)	0.04	0.00-0.18	µmol/L
Adipylcarnitine (C6DC)	0.07	0.00-0.25	µmol/L
Octanoylcarnitine (C8)	0.22	0.00-0.23	µmol/L
Octenoylcarnitine (C8:1)	0.04	0.00-0.22	µmol/L
Decanoylcarnitine (C10)	0.13	0.01-0.26	µmol/L
Decenoylcarnitine (C10:1)	0.04	0.00-0.18	µmol/L
Decadienoylcarnitine (C10:2)	0.02	0.00-0.08	µmol/L
Dodecanoylcarnitine (C12)	0.04	0.00-0.42	µmol/L
Dodecenoylcarnitine (C12:1)	0.03	0.00-0.32	µmol/L
Tetradecanoylcarnitine (C14)	0.08	0.01-0.50	µmol/L
Tetradecenoylcarnitine (C14:1)	0.04	0.01-0.37	µmol/L
Tetradecadienoylcarnitine (C14:2)	0.01	0.00-0.05	µmol/L
3-Hydroxy-tetradecanoylcarnitine (C14OH)	0.01	0.00-0.05	µmol/L
Hexadecanoylcarnitine (C16)	0.89	0.4-6.0	µmol/L
Hexadecenoylcarnitine (C16:1)	0.05	0.02-0.44	µmol/L
3-Hydroxy-hexadecenoylcarnitine (C16:1OH)	0.02	0.00-0.13	µmol/L
3-Hydroxy-hexadecanoylcarnitine (C16OH)	0.00	0.00-0.08	µmol/L
Octadecanoylcarnitine (C18)	0.50	0.18-1.70	µmol/L
Octadecenoylcarnitine (C18:1)	1.57	0.29-2.95	µmol/L
3-Hydroxy-octadecenoylcarnitine (C18:1OH)	0.00	0.00-0.08	µmol/L
Octadecadienoylcarnitine (C18:2)	0.35	0.02-0.60	µmol/L
3-Hydroxy-octadecanoylcarnitine (C18OH)	0.00	0.00-0.06	µmol/L

Multiple studies have shown a strong connection between anti-NMDA receptor encephalitis and viral infections, including herpes simplex virus, Japanese encephalitis virus, and even COVID-19. Therefore, real-time polymerase chain reaction (PCR) for viruses was performed in this case [4].

First treatment

Based on the antibody result and the typical regression, he was diagnosed with autoimmune encephalitis, for which we decided to initiate multimodal immunotherapy. As first-line immunotherapy for anti-NMDA receptor encephalitis consists of high-dose intravenous methylprednisolone, either alone or in combination with intravenous immunoglobulin (IVIG), the patient was treated with intravenous methylprednisolone (Solu-Medrol) at a dose of 30 mg/kg/day for five consecutive days, from April 9 to April 13 [5].

Clinical progression

On April 16, he experienced a transient deterioration with increasing seizures, fatigue, inability to sit up without support, and decreased mobility.

Examination on April 16 revealed that the patient was awake but had limited communication. There were no seizures during the examination. He had hypotonia. Deep tendon reflexes were preserved. Meningeal signs were positive/negative.

This is a very typical clinical presentation that may occur in patients with autoimmune encephalitis when corticosteroid therapy alone is insufficient to control the antibody-mediated inflammatory process. Therefore, given the lack of response to steroids and the worsening clinical condition, we decided to escalate immunotherapy with intravenous immunoglobulin at a total dose of 2 g/kg/5 days, administered from April 21 to April 24 [5].

Outcome and follow-up

The response after IVIG infusion was dramatic, with approximately 70%-80% recovery. Cognitively, the child became more alert, turned his head when called, and began following commands and understanding speech. Motor function improved, and the patient was able to move normally and had no focal neurological signs. However, he still had mild movement disorder symptoms in his right hand.

At the time of discharge, his speech skills still required more time to fully recover. However, we observed significant improvement in both motor function and cognitive behavior, indicating that the decision to initiate immunotherapy was appropriate. A follow-up appointment was scheduled, and the patient’s family were also advised regarding the next dose of immunotherapy, which was planned for the next month.

## Discussion

To begin with, first, we found no evidence supporting a causal relationship between the Japanese encephalitis vaccination administered one day before symptom onset and the patient’s subsequent clinical condition, given the extremely short interval between vaccination and symptom onset. Japanese encephalitis vaccination does not typically trigger anti-NMDA receptor IgG synthesis [6].

A prospective study by Liu et al., published in the Journal of the Neurological Sciences in May 2021, evaluated 31 patients with Japanese encephalitis. All patients were negative for anti-neuronal surface antibodies in both serum and cerebrospinal fluid at disease onset. However, during the convalescent phase, five patients subsequently developed autoimmune encephalitis-associated antibodies: two developed anti-NMDA receptor antibodies, one developed anti-GABA receptor antibodies, and two developed antibodies against unidentified neuronal surface antigens [7]. These findings suggest that autoimmune encephalitis may emerge as a post-infectious complication of JE and typically develops during the convalescent phase rather than immediately after antigen exposure.

Second, this case emphasizes that a normal brain MRI does not exclude anti-NMDA receptor encephalitis. Previous studies have reported that up to 50% of patients with anti-NMDAR encephalitis may have normal brain MRI findings during the early stages of the disease. Likewise, EEG and cerebrospinal fluid analyses may initially be nondiagnostic. Therefore, normal MRI, EEG, and CSF findings should not preclude consideration of anti-NMDAR encephalitis when the clinical presentation is suggestive.

A prospective study by Huong et al. conducted at a level 1 children’s hospital in Vietnam enrolled 164 children with a clinical diagnosis of encephalitis. The identified etiologies included anti-NMDA receptor encephalitis in 23 (14%) cases, Japanese encephalitis virus infection in 14 (8.5%) cases, and herpes simplex virus encephalitis in 4 (2.4%) cases. Notably, anti-NMDA receptor encephalitis was more common than Japanese encephalitis, which remains the leading cause of infectious encephalitis in many parts of Southeast Asia [8]. These findings might change our approach to this clinical setting, in which we should consider earlier autoimmune causes, even in a tropical country. Increased awareness and timely antibody testing may facilitate earlier diagnosis and treatment, ultimately improving patient outcomes.

Third, the abrupt loss of previously acquired language skills represented a critical clinical warning sign. Importantly, clinicians should distinguish developmental delay from developmental regression. Developmental delay refers to slower acquisition of milestones, whereas developmental regression is characterized by the loss of previously acquired skills. In children with a history of mild developmental delay, sudden loss of language or motor abilities should raise concern for an acute neurological process and the importance of early investigation for autoimmune encephalitis, including neuronal antibody testing.

Finally, the child was initially diagnosed with epilepsy and only later developed language regression and loss of ambulation. This clinical course highlights that anti-NMDA receptor encephalitis may initially present with seizures before the full clinical setting becomes apparent [9]. Previous studies have shown that neurological manifestations, including seizures, movement disorders, and focal neurological deficits, are often more prominent than psychiatric symptoms at disease onset in pediatric patients [10]. Early recognition and timely initiation of immunotherapy are associated with improved neurological outcomes and lower relapse rates.

In children, neurological manifestations such as seizures, movement disorders, and focal neurological deficits are more prominent at initial presentation than psychiatric or behavioral symptoms. When psychiatric symptoms do occur, they often manifest as temper tantrums, aggression, agitation, and rarely psychosis [11]. Prompt diagnosis and early treatment can lead to improved outcomes and decreased relapses.

## Conclusions

A key lesson from this case is that the diagnosis of anti-NMDA receptor encephalitis relies on clinical suspicion. When provisional investigations such as MRI, EEG, and CSF analysis are inconclusive, clinicians should not dismiss the possibility of autoimmune encephalitis. When autoimmune encephalitis is suspected, immunotherapy should be initiated immediately. Early immunotherapy can lead to meaningful neurological recovery, even when diagnosis is delayed. Patients who do not respond adequately to corticosteroid therapy may improve after escalation to intravenous immunoglobulin, plasma exchange, or combination therapy. In children with suspected autoimmune encephalitis, timely recognition, antibody testing, treatment escalation, and follow-up planning are essential to reduce long-term neurological disability.

## References

[1] Graus F, Titulaer MJ, Balu R (2016). A clinical approach to diagnosis of autoimmune encephalitis. Lancet Neurol.

[2] Guasp M, Dalmau J (2025). Autoimmune encephalitis. Med Clin North Am.

[3] Iizuka T, Sakai F, Ide T (2008). Anti-NMDA receptor encephalitis in Japan: long-term outcome without tumor removal. Neurology.

[4] Yan Hung SK, Hiew FL, Viswanathan S (2019). Anti-NMDAR encephalitis in association with herpes simplex virus and viral and bacterial zoonoses. Ann Indian Acad Neurol.

[5] Ma J, Han W, Jiang L (2020). Japanese encephalitis-induced anti-N-methyl-d-aspartate receptor encephalitis: a hospital-based prospective study. Brain Dev.

[6] Stingl C, Cardinale K, Van Mater H (2018). An update on the treatment of pediatric autoimmune encephalitis. Curr Treatm Opt Rheumatol.

[7] Liu B, Liu J, Sun H (2021). Autoimmune encephalitis after Japanese encephalitis in children: a prospective study. J Neurol Sci.

[8] Huong NH, Toan ND, Thien TB (2024). In children, N-methyl-D-aspartate receptor antibody encephalitis incidence exceeds that of Japanese encephalitis in Vietnam. Open Forum Infect Dis.

[9] Hardy D (2022). Autoimmune encephalitis in children. Pediatr Neurol.

[10] Ursitti F, Roberto D, Papetti L (2021). Diagnosis of pediatric anti-NMDAR encephalitis at the onset: a clinical challenge. Eur J Paediatr Neurol.

[11] Titulaer MJ, McCracken L, Gabilondo I (2013). Treatment and prognostic factors for long-term outcome in patients with anti-NMDA receptor encephalitis: an observational cohort study. Lancet Neurol.

